# High reclassification rate of height-indexed left atrial volume in obese and overweight patients with cardiac pathologies in daily clinical practice

**DOI:** 10.1038/s41598-026-37809-z

**Published:** 2026-01-30

**Authors:** Edmundo José Nassri Câmara, Flávia R. do Prado Valladares, Marcus Ribeiro de O. Santana, Adriano da Cunha Gomes, Leonardo Soares Carvalho, Sérgio de Souza Silva Buruaem, Alber Oliveira Silva, Ananda Sousa Silva, Aureo Bomfim Teixeira, Heitor Delfino da Silva, Najla Yasmin Felix, Reginaldo Vital da Silva Neto

**Affiliations:** 1 Federal University of Bahia, Salvador-Bahia, Brazil; 2 Department of Echocardiography, Hospital Ana Nery, Salvador-Bahia, Brazil; 3 Fellow of Echocardiography, Hospital Ana Nery, Salvador-Bahia, Brazil

**Keywords:** Cardiology, Diseases, Medical research, Risk factors

## Abstract

We sought to investigate the frequency of left atrial (LA) size reclassification in obese and overweight patients with heart disease, adjusting LA volume (LAvol) for height rather than BSA. Obesity is associated with cardiovascular diseases, heart failure with preserved ejection fraction (HFpEF) and atrial fibrillation, among others. Indexing LAvol in obese patients by BSA may misclassify LAvol and the diagnosis of LA dilation. We consecutively studied 253 patients, mean age of 64.3 ± 15.1 years, 146 men (57.7%) with cardiac pathologies. LAvol was measured by the biplane Simpson’s rule. We compared LAvol indexed by BSA, height and height², prevalence and degree of LA dilation among normal weight (< 25 kg/m²), overweight (≥ 25 < 30 kg/m²) and obese (≥ 30 kg/m²) patients. There were 111 (44%) normal weight, 59 (23%) obese and 83 (33%) overweight patients. Overall, the frequency of LA dilation was 58%, 64.2% and 63.8%, indexed by BSA, height and height², respectively. In obese patients these figures were 46%, 65% and 66.6%. Twelve of 30 (40%) obese and 7 of 33 (21%) overweight patients were reclassified from non-dilated to dilated LA when indexed either by height or height². Half (50%) of obese patients were upgraded by one grade, and 37.5% by two grades. Obesity, BMI ≥ 27.5 kg/m² and atrial fibrillation were independent predictors of reclassification. Reclassification of LAvol indexed for height in obese or overweight patients with heart disease is very high. It seems appropriate to use LAvol indexed for height in these patients.

## Introduction

Increased left atrial volume (LAvol) is a risk marker for atrial fibrillation, stroke, and heart failure, and it has been correlated with increased cardiovascular mortality^1^. International guidelines and clinical practice recommend that LAvol be indexed to body surface area (BSA). However, in obese individuals there is a tendency to overcorrection the left atrium (LA) size, often placing individuals with an enlarged left atrium as normal, after indexing by a high BSA. This is an important problem, because obesity is associated with atrial fibrillation that appears to be mediated by LA dilation^2–4^ and it is recognized as a risk factor for heart failure (HF), especially for HF with preserved ejection fraction (HFpEF)^5,6^. The prevalence of obesity is increasing worldwide^7–9^, it is likely that prevalence of obesity related AF will increase as well. BSA is an isometric measure, which assumes that LA size has a linear relationship to body size. However, organ size (i.e., heart size) and body size do not grow proportionally; in other words, their relationship is nonlinear. Many parameters can be indexed for height, especially in obese individuals.

Alternative forms of scaling, including allometric size variable such as height raised to an exponent, may help address this issue. Recently we reported the normal values for the LAvol indexed to height in a Brazilian population^10^. We found that more than 20% of the obese apparently normal patients had increased LAvol when indexed for height. By the contrary, in the normal weight group of patients none had increased LAvol when indexed by height.

Therefore, we aimed to investigate, in our daily practice in an echocardiography laboratory, the proportion of obese patients with cardiac pathologies who would be reclassified for LA dilation using height-indexed volume instead of BSA.

## Methods

We studied a total of 275 patients who underwent echocardiography at our service in Hospital Ana Nery, Salvador-Bahia, Brazil. The participants of this study were directly and consecutively recruited for the study. Informed consent was obtained from all subjects and/or their legal guardian(s). The protocol of the study was approved by the Research Ethics Committee (Comitê de Ética e Pesquisa) of the Hospital Ana Nery in November 2021. Only patients with cardiac pathologies, such as cardiomyopathies, heart failure, hypertensive cardiovascular disease, ischemic heart disease, atrial fibrillation, and valvular diseases other than primary mitral valve disease, were included. Patients with moderate to severe primary mitral valve disease and patients with normal echocardiograms were not included. Twelve patients were excluded, five due to inadequate echocardiographic windows and seven due to lack of data. The remaining 253 patients constituted our study cohort. LAvol was obtained by the Simpson’s biplane method of disks using apical four chamber and two chamber views at ventricular end-systole, according to the recommendations from the American Society of Echocardiography and the European Association of Cardiovascular Imaging^11^. The anteroposterior diameter of the left atrium was measured using two-dimensional imaging in the parasternal longitudinal plane during end-of-systole, in accordance with the recommendations of the American Society of Echocardiography and the European Association of Cardiovascular Imaging^11^. In addition, LV dimensions and wall thickness, left ventricular mass and aortic root diameter were also measured using a two-dimensional image in the parasternal longitudinal plane^11^. All methods were performed in accordance with the relevant guidelines and regulations. According to body mass index (BMI) patients were classified as obese (BMI ≥ 30 Kg/m²), overweight (≥ 25 < 30 Kg/m²) or normal weight (< 25 Kg/m²). To the diagnosis of LA dilation we used the normal values and the cutoffs described in our population in normal non obese individuals^10^. The cutoffs were: LAvol/BSA 34.2 ml/m², LAvol/height 35.0 ml/m and LAvol/height² 21.6 ml/ht². These values ​​are very close to those described in other populations and in guidelines^11,12^ and were observed in our population, in people of normal weight and without heart disease^10^.

### Statistical analysis

Continuous variables are described as mean ± standard deviation (SD) and were compared using Student T test when a normal distribution was present, otherwise, a nonparametric test was used, such as Mann- Whitney. One-way analysis of variance or a nonparametric test (Kruskal-Wallis) was used when more than two groups were compared. Categorical data were compared by means of the chi-square test. We used the ROC curve to find the best BMI cutoff point to separate patients who would change classification from normal to dilated LA after indexing for height. We used binary logistic regression analyses to investigate factors that were independently associated with LA reclassification from normal to dilated using LAvol indexing by height in addition to BSA. Multiple binary logistic regression with the covariates age, sex, obesity, BMI cutoff point ≥ 27 kg/m², atrial fibrillation, hypertensive heart disease, and LVEF were done to determine predictors of reclassification for dilated LA in univariate analysis. These variables were incorporated into a multivariate binary logistic regression analysis using the Forward Stepwise (Condicional) method to identify independent predictors of LA reclassification indexing by height. To avoid collinearity, obesity and BMI ≥ 27.5 kg/m² were tested separately. All statistical analysis were performed using IBM SPSS statistics version 25. A P-value < 0.05 was considered significant.

## Results

Baseline clinical and echocardiographic characteristics of the patients are in Table 1.

The mean age was 64.3 ± 15.1, there were 146 males (57.7%) and 107 females (42.3%), 111 (44%) normal weight, 59 (23%) obese, 83 (33%) overweight patients. There were few differences between the groups, such as the fact that obese patients were younger (60.1 ± 13.2 y.o.) comparing to normal weight (66.7 ± 17 y.o.) – *p* = 0.018 and that it was observed a predominance of females in this group (*p* = 0.001). Obesity was less frequent in patients with atrial fibrillation while it was more frequent in those with hypertensive heart disease.

The indexed LAvol for BSA was smaller in obese and overweight groups, meanwhile there were no differences when the LAvol was indexed for height (Table 2).

**Fig. 1:**
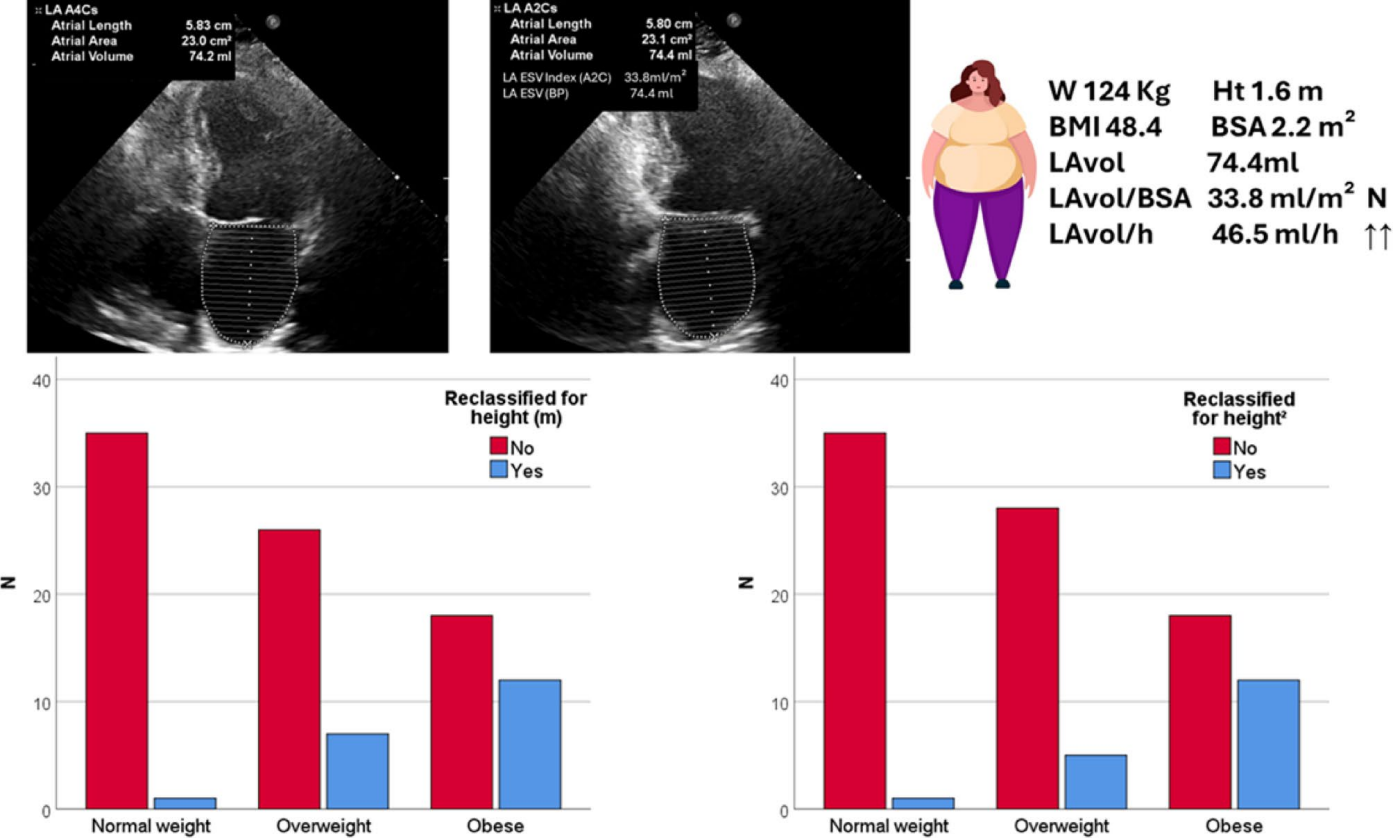
Reclassification of LA enlargement according to weight category using ht(m) and ht^2^ indexing insteady of BSA. Reference^10^ for the normal values and cutpoints of severity.

**Fig. 2:**
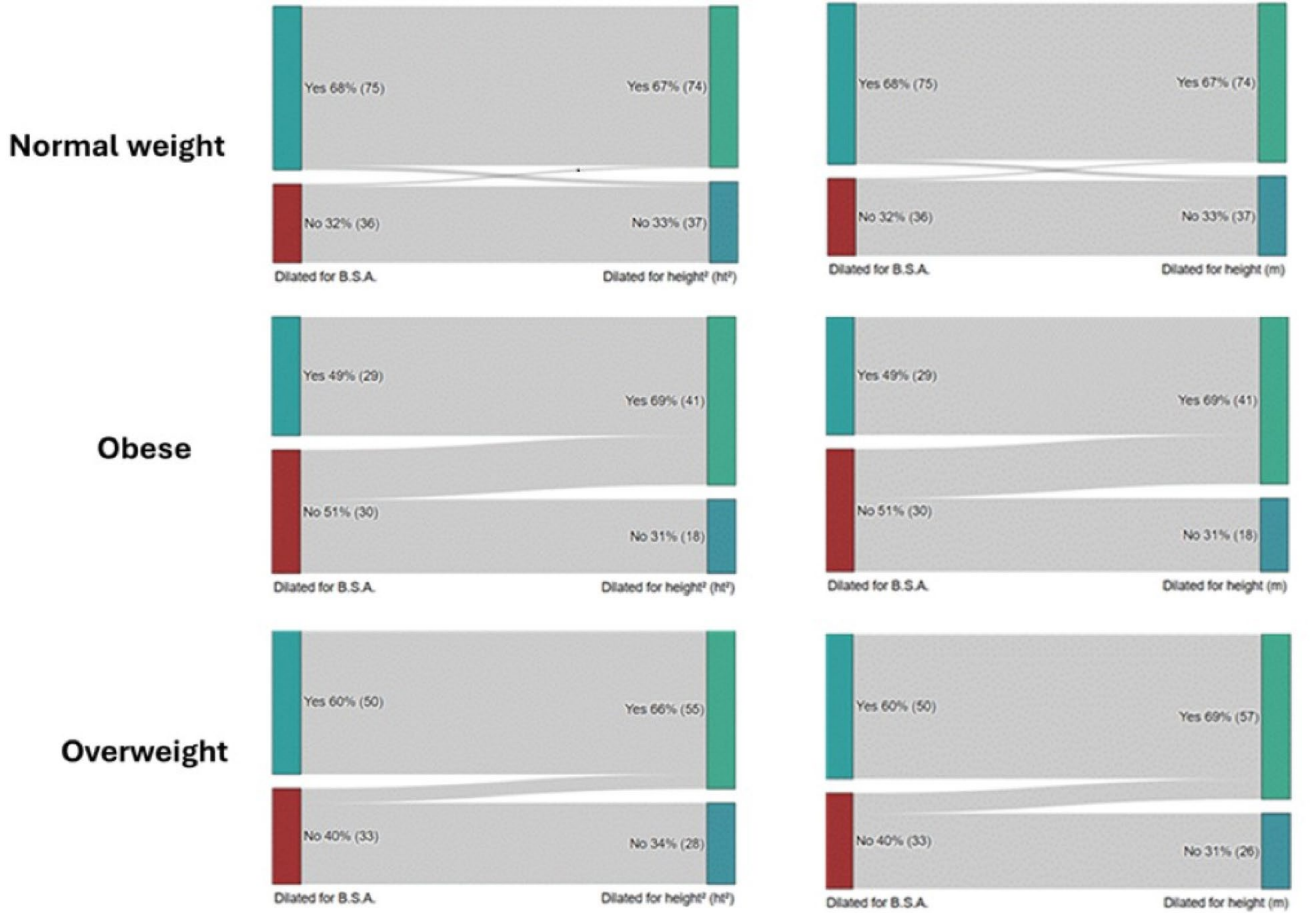
Reclassification of LA enlargement according to weight category using ht(m) and ht^2^ indexing instead of BSA. Reference^10^ for the normal values and cutpoints of severity.

Regarding the diagnosis of LA dilation, 156 (58%) patients were diagnosed by LAvol/BSA indexing, 174 (64.2%) by LAvol/ht indexing and 173 (63.8%) by LAvol/ht² indexing. Twenty of 115 (17%) patients were reclassified from non-dilated to dilated by indexing LAvol/ht and 19 of 115 (16.5%) patients were reclassified by indexing LAvol/ht². In the obesity group, only 29 (46%) patients were diagnosed by LAvol/BSA indexing, while 41 (65%) by LAvol/ht indexing and 42 (66.6%) by LAvol/ht² indexing. On the other hand, analyzing the 30 obese patients with normal LAvol by BSA, 12 of them (40%) were reclassified as having LA dilation when indexing LAvol by height and also by height². In contrast, only 01 of 40 patients with normal weight and normal LAvol/m² were reclassified by indexing LAvol to height as well as to height² (Table 3; Figs. 1 and 2). All obese or overweight patients with LA dilation by indexing for BSA were identified indexing by both for height or for height².

In normal weight patients only 2 of 76 (2.6%) with the diagnosis of LA dilation by indexing LAvol/BSA were not identified by indexing LAvol either for height or height².

Regarding the severity of the LA dilation, we used the cutoffs of > 42 ml/m², > 48 ml/m² indexing by BSA according to ASE/EACI recommendations^12^, > 44 ml/m, > 48.5 ml/m indexing by height, > 27 ml/ht² and > 30 ml/ht² indexing by height², to classified as moderate and severe LA dilation, corresponding to four and five standard deviation of the normal values we described in our population^10^. Half (50%) of obese patients were upgraded by one grade, and 37.5% by two grades indexing by height or by height² (Table 3). This did not occur in normal weight patients. In addition, all obese patients classified as severe dilation indexing by BSA were also classified as severe dilation indexing by height or by height².

**Table 1:** Baseline demographic, clinical and echocardiographic data.

	All patients *N* = 253	Normal weight *N* = 111	Overweight *N* = 83	Obese *N* = 59	*P*
Age (y.o)	64.4	66.9	64.2	60.1	0.018
Sex (M/F)	146/107	67/44	57/26	22/37	0.001
Weight (Kg)	70.8	57.6	73.7	91.5	0.000
Height (cm)	162.6	161.86	164.35	161.44	0.090
BSA (m^2^)	1.75	1.61	1.80	1.95	0.000
B.M.I. (Kg/m^2^)	26.2	21.9	27.2	35.0	0.000
Atrial fibrillation	92 (36%)	49 (44%)	30 (36%)	13 (22%)	0,04
Hypertensive heart disease	44 (17%)	14 (12.6%)	13 (15.6%)	17 (29%)	0,03*
Ischemic heart disease	81 (32%)	31 (28%)	30 (36%)	20 (34%)	0.218
Cardiomyopathy	23 (9%)	12 (11%)	7 (8%)	4 (8%)	0.400
Cardiac surg/intervention	45 (18%)	15 (13.5%)	17 (20%)	13 (22%)	0.116
Miscellaneous	45 (18%)	23 (21%)	14 (17%)	8 13.5%)	0.281
LVEDD (mm)	49.6	49.0	49.8	50.4	0.543
LV mass (g)	188.4	177.8	192.3	203.1	0.068
LV mass indexed (g/m^2^)	107.8	110.9	106.5	103.7	0.484
LV ejection fraction	58.4	56.6	59.2	60.6	0.174
Mean E/E’	11.1	11.3	11.1	10.9	0.855
LAvol (ml)	75.7	80.4	71.2	73.1	0.251
LAvol ind (ml/m^2^)	43.9	50.6	39.5	37.6	0.013**

* Obese versus normal weight, ** Obese or overweight versus normal weight.

**Fig. 3:**
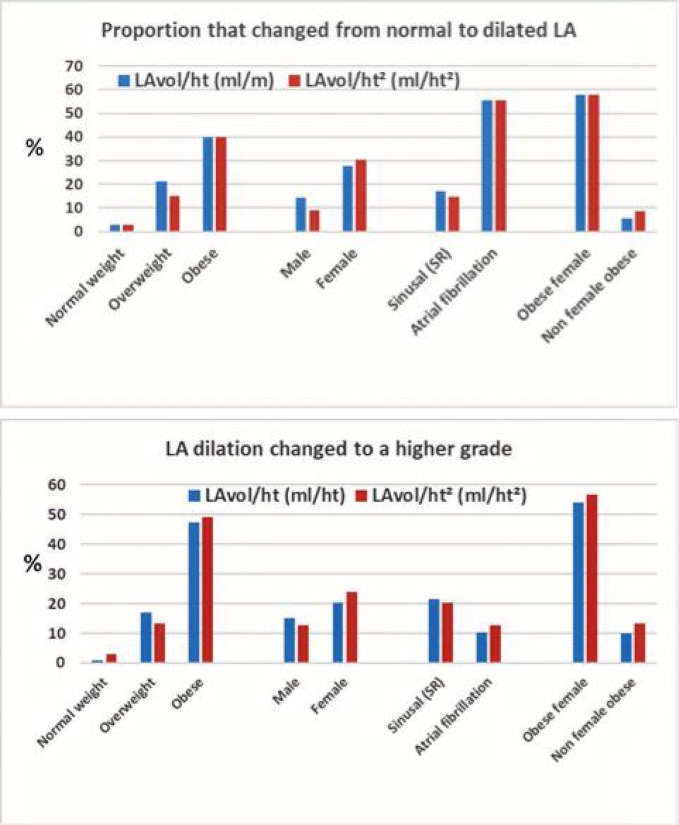
Factors that most contributed to the reclassification of the LA volume.

Factors that most contributed to the reclassification were female sex, overweight/obesity and atrial fibrillation (Fig. 3). Using the ROC curve, the best cutoff point at which height indexing would begin to have a greater impact, compared to BSA, for identifying patients with left atrial dilation was 27.5 Kg/m². Obese women (OR 14.7, 95% CI 3.3–65.3) and patients with atrial fibrillation rhythm (OR 6.1, 95% CI 1.46–25.3) were the most affected groups. In a multivariate analysis, obesity (OR 6.8, 95% CI 2.2–20.6, *p* = 0.001), BMI ≥ 27.5 Kg/m² (OR 8.2, 95% CI 2.2–30.6, *p* = 0.002) and atrial fibrillation (OR 11.0, 95% CI 2.0–59.0, *p* = 0.005) were independent predictors of LA reclassification, while sex (OR 2.6, 95% CI 0.7–9.6, *p* = 0.155), age (OR 1.001, 95% CI 0.95–1.05, *p* = 0.96), hypertensive heart disease (OR 1.77, 95% CI 0.36–8.8, *p* = 0.485) and ejection fraction (OR 1.002, 95% CI 0.95–1.05, *p* = 0.948) were not. In a univariate analysis, sex was associated with LA reclassification (OR 4.4, 95% CI 1.4–13.6, *p* = 0.010).

**Table 2:** Left atrial dimension and volume according to the weight.

				95% CI		P between groups	
		Mean	SD	Min	Max	Anova	K-Wallis
LA diam (mm)	Eutrophy	41.8	8.4	40.2	43.4	0.9	0.7
	Overweight	42.3	7.2	40.7	43.8		
	Obesity	42.1	6.8	40.3	43.8		
LAvol (ml)	Eutrophy	80.4	53.1	70.4	90.4	0.25	0.88
	Overweight	71.2	27.3	65.2	77.1		
	Obesity	73.1	25.7	66.4	79.9		
LAvol/(BSA) (ml/m^2^)	Eutrophy	50.6	34.3	44.1	57.0	0.001	0.013
	Overweight	39.5	15.0	36.2	42.7		
	Obesity	37.6	13.0	34.2	41.0		
LAvol/height (ml/m)	Eutrophy	49.7	32.9	43.5	55.9	0.19	0.68
	Overweight	43.2	16.3	39.7	46.8		
	Obesity	45.3	15.6	41.2	49.3		
LAvol/height^2^ (ml/ht^2^)	Eutrophy	30.8	20.6	29.9	34.7	0.14	0.49
	Overweight	26.3	10.0	24.2	28.5		
	Obesity	28.1	9.7	25.6	30.7		

**Table 3:** Grade of left atrial dilation in obese patients classified by BSA, height and height^2^.

By BSA	By height				By height^2^			
	Mild	Mod.	Severe	Normal	Mild	Mod.	Severe	Normal
Mild	1	4	3	0	1	4	3	0
Mod.	0	1	9	0	0	0	10	0
Severe	0	0	11	0	0	0	11	0
Normal	8	4	0	18	10	2	0	18

## Discussion

In this study, in patients with heart disease, we show a very high frequency of left atrial size reclassification in obese and overweight patients, from non-dilated to dilated LA, when volume was indexed by height rather than body surface area. Furthermore, it was also observed that the severity of LA dilation changed from mild to moderate/severe and even from normal to mild or moderate. In obese patients, 40% migrated from non-dilated to dilated LA. In overweight (non-obese) patients, 21% changed from normal to dilated. On the other hand, in normal weight patients, only one of them changed from non-dilated to dilated when indexed by height or by height².

The current study highlights the importance of indexing the LA volume by height instead of indexing by BSA in obese patients. The results were very similar Indexing by height or by height², however the number of patients may not be sufficient to confirm this.

Obesity is known to be associated with a higher prevalence of conditions that are mediated by LA enlargement such as LV diastolic dysfunction, HFpEF and atrial fibrillation^2,4,5,7,13,14^. It has been described as the strongest predictor of LA size in patients with hypertension^15^. When LA size was indexed based on methods that were not weight dependent, a higher prevalence of LA enlargement was noted in obese patients, furthermore, obesity was the main risk factor for left atrial enlargement during aging, even stronger than hypertension^16,17^. In our study, which we report now, obesity, BMI ≥ 27.5 Kg/m² and atrial fibrillation were strongly and independently associated with reclassification for LA dilation, when LAvol was indexed for height instead of BSA. Similarly, but in a population with moderate to severe obesity (BMI ≥ 35 kg/m2), Aga Y et al. reported a significantly greater frequency of LA dilation when LAvol was indexed for height² instead of BSA (54.2% vs. 18.3%) and that significantly more patients with LA dysfunction were correctly identified by LAvol/ht² than by LAvol/BSA^18^.

The impact of indexing left atrial volume by height on prognosis and future events, including death, was well demonstrated by Davis EF et al. in 2022. This is an important longitudinal study with 17,454 individuals, proving that left atrial dilation based on indexing by height or Ht² showed better predictive performance in the studied population, and better overall predictive performance in all overweight and obese populations, while standard indexing by body surface area (BSA) was not discriminatory for predicting mortality in patients with severe obesity^19^. Another important aspect of this study, which reinforces our findings, was that patients whose left atrium was reclassified from normal to dilated had an increased risk of mortality (*P* < 0.001) and cardiovascular events (*P* < 0.001) in all BMI categories.

LA function and volume are increasingly being used for evaluating LV diastolic function and to the diagnosis of elevated left heart filling pressures. According to guidelines recommendations, LA volume indexed by BSA is one of the criteria for the diagnosis of diastolic dysfunction with elevated filling pressures^20,21^. and, consequently, for the diagnosis of HFpEF (heart failure with preserved ejection fraction)^22^. According to our data, in many obese patients, indexing LA volume for BSA may lead to a big error, the real LA size will be overcorrect, which may negatively impact the clinical and therapeutic approach, and the prognosis of these patients, including mortality, as already well demonstrated^19^.

A cutoff of 29 ml/m² has been suggested for the diagnosis of LA dilation in obese patients using LAVol/BSA^23^. However, this does not take into account the degree of obesity, whereas height is an independent and patient-specific parameter.

LA volume tends to be higher when measured by 3D echocardiography compared with 2D indexing method. Therefore, we suppose that the normality values for LA volume by 3D echocardiography should also be indexed for height in obese and overweight individuals.

The assessment of left atrial size should be done by measuring the volume, preferably using a 3D method if possible. Diameter of the left atrium, especially the anteroposterior diameter, is significantly influenced by chest wall conformation, is limited by the physical constraints of the spine and the sternum, it is not clinically relevant^24^. In obesity, the increased anteroposterior diameter of the thorax may be one of the reasons for the increased size of the left atrium^25^.

Our study has some limitations. The cohort is relatively small, especially the obese subgroup (*n* = 59). Caution should be exercised by generalizing the results, although our findings are consistent with studies in other populations. Measurements were performed by only one echocardiographer, so the interobserver variability was not estimated. Since the same volume obtained was indexed for BSA and for height, we are not comparing methods of measurements, it probably did not interfere with the conclusions. Another limitation is that, as this is a cross-sectional study, we do not have a comparison between the two indexing methods as predictors of future events. This falls beyond the scope of the current study, but it has been already well demonstrated by Davis EF et al. in 2022, an important longitudinal study^19^.

In conclusion, we strongly recommend using left atrial volume indexed by height (height or height²) rather than using left atrial volume indexed by BSA in obese patients as well as in those who are overweight.

## Data Availability

The datasets used and/or analysed during the current study available from the corresponding author on reasonable request.
